# The transcriptional coactivator PGC1*α* protects against hyperthermic stress via cooperation with the heat shock factor HSF1

**DOI:** 10.1038/cddis.2016.22

**Published:** 2016-02-18

**Authors:** L Xu, X Ma, A Bagattin, E Mueller

**Affiliations:** 1 Genetics Development and Disease Branch, National Institute of Diabetes, Digestive and Kidney Diseases, National Institutes of Health, Bethesda, MD, USA

## Abstract

Heat shock proteins (HSPs) are required for the clearance of damaged and aggregated proteins and have important roles in protein homeostasis. It has been shown that the heat shock transcription factor, HSF1, orchestrates the transcriptional induction of these stress-regulated chaperones; however, the coregulatory factors responsible for the enhancement of HSF1 function on these target genes have not been fully elucidated. Here, we demonstrate that the cold-inducible coactivator, PGC1*α*, also known for its role as a regulator of mitochondrial and peroxisomal biogenesis, thermogenesis and cytoprotection from oxidative stress, regulates the expression of HSPs *in vitro* and *in vivo* and modulates heat tolerance. Mechanistically, we show that PGC1*α* physically interacts with HSF1 on HSP promoters and that cells and mice lacking PGC1*α* have decreased HSPs levels and are more sensitive to thermal challenges. Taken together, our findings suggest that PGC1*α* protects against hyperthermia by cooperating with HSF1 in the induction of a transcriptional program devoted to the cellular protection from thermal insults.

Heat shock proteins (HSPs) are members of an evolutionary conserved family of proteins whose expression increases in response to a variety of different metabolic insults.^1^ These factors are classified primarily according to their molecular weight as HSP40, HSP60, HSP70, HSP90, HSP100 and small HSPs.^2^ HSP40s, also known as Dnaj proteins, are co-chaperones assisting chaperones such as HSP70 and HSP90 in their functions.^1, 3^ In stressful conditions, such as during heat shock, oxidative stresses or hypoxia, HSPs function by stabilizing unfolded or misfolded peptides, restraining protein aggregation through co-chaperone-mediated cycles of ATP hydrolysis and ADP release and by binding with chaperones until the native protein conformation is restored. When the refolding process fails, chaperones also assist the degradation of the aberrant/misfolded proteins via the ubiquitin-proteasome system.^4^ HSPs and their cofactors have been shown to be responsible for inhibiting both apoptotic and necrotic pathways during cell death.^5^ These molecular chaperones function as protectors of the proteome and have central roles in the physiology of aging and neurodegeneration.^6, 7^

Hyperthermia, the classic inducer of HSPs, leads to heat stroke and can arise in genetically predisposed subjects with mutations in the ryanodine receptor gene^8^ and in subjects with adverse reactions to drugs such as selective serotonin reuptake inhibitors, monoamine oxidase inhibitors, tricyclic antidepressants and volatile anesthetic gases.^9^ The family of the 70 kilodalton HSPs (HSP70s) includes 13 members in humans widely expressed within all the subcellular compartments.^4, 10^ Among them, HSC70 (HSPA8) is a constitutively expressed chaperone, while HSPA5 (GRP78), localized to the endoplasmic reticulum, and HSPA9 (mtHSP70), present in the mitochondrial compartment, are stress-induced. HSP70 paralogs HSPA1A, HSPA1B and HSPA1L have been shown to be involved in the refolding of damaged or defective proteins, in their targeting for degradation and for directly inhibiting apoptosis, thus ultimately protecting cells from thermal and oxidative stress.^10^

The transcriptional activation of the heat shock response is orchestrated by the heat shock factor 1 (HSF1).^1, 11^ HSF1 exists as an inactive monomer in a complex with Hsp40, Hsp70 and Hsp90. Upon physiological stresses resulting from hyperthemia or oxidative insults, HSF1 is released from the chaperone complex and, upon trimerization, it translocates into the nucleus and binds to heat shock-responsive elements (HSEs) present on target gene promoters to initiate their transcriptional activation.^12^ It has been shown that HSF1-null fibroblasts are more susceptible to heat-induced apoptosis because of the absence of HSP induction^13^ and that HSF1-null mice fail to raise HSP levels in response to thermal insults,^14^ suggesting critical roles of HSF1 in the regulation of HSPs in stress defense. In addition to the transition of active and inactive states of HSF1 by association with HSPs, previous studies have also reported that the activation and attenuation of HSF1 activity is achieved through extensive posttranslational modifications, including phosphorylation, sumoylation and acetylation.^12^ Although HSF1 has been implicated in counteracting cell stress and is critical in cancer biology, aging and neurodegenerative diseases, little is known about the regulatory molecules and complexes that modulate its activity.^1, 15^

Peroxisome proliferator-activated receptor *γ* coactivator 1*α* (PGC1*α*) is a cold-inducible coactivator that controls mitochondrial and peroxisome biogenesis and energy metabolism.^16, 17^ In addition to these functions, it has been shown that PGC1*α* has dual roles in maintaining cellular and tissue homeostasis by increasing mitochondrial activity and preventing oxidative stress through the induction of reactive oxygen species (ROS)-detoxifying enzymes.^18^ Studies in wild-type (WT) and PGC1*α*-null mice have demonstrated that absence of PGC1*α in vivo* is associated with striatum lesions and increased dopaminergic cell death and oxidative damages upon treatment with MPTP, a compound known to induce neourotoxicity in animal models and humans.^18, 19^ Despite the clear role of PGC1*α* in ROS protection, less is known about the function of PGC1*α* in response to other types of stressors. Recently, the results of genome-wide sequencing of PGC1*α*-binding sites in hepatocytes have suggested the potential occupancy of PGC1*α* at the heat shock elements (HSE) present at promoters of HSPs.^20^ In addition, recent data from our laboratory have demonstrated the physical interaction and co-occupancy of PGC1*α*-HSF1 at the HSE present on the promoter of PGC1*α*.^21^ On the basis of these reports, we hypothesized the potential cooperation of PGC1*α* and HSF1 in regulating HSPs under stress conditions and assessed the possible role of PGC1*α* in the protection from hyperthermic stress and its pathological consequences in animals.

Here, we report that PGC1*α* is sufficient to induce HSPs both *in vitro* and *in vivo* and that it interacts with HSF1 at the HSE present at the promoter of HSP70 after heat shock stimuli. Furthermore, we show that cells with deficiency in PGC1*α* have reduced HSPs levels and that are more sensitive to hyperthermia-induced apoptosis. In addition, PGC1*α* knockout (KO) mice show decreased ability to adapt to changes in temperatures as demonstrated by the increased apoptosis in kidney and liver after thermal challenge. Restoration of PGC1*α* levels in PGC1*α* KO cells and livers rescued heat tolerance. Overall, our results establish PGC1*α* as a new critical orchestrator of thermotolerance in cooperation with HSF1.

## Results

### PGC1*α* is sufficient to induce HSPs *in vitro*

**Figure 1:**
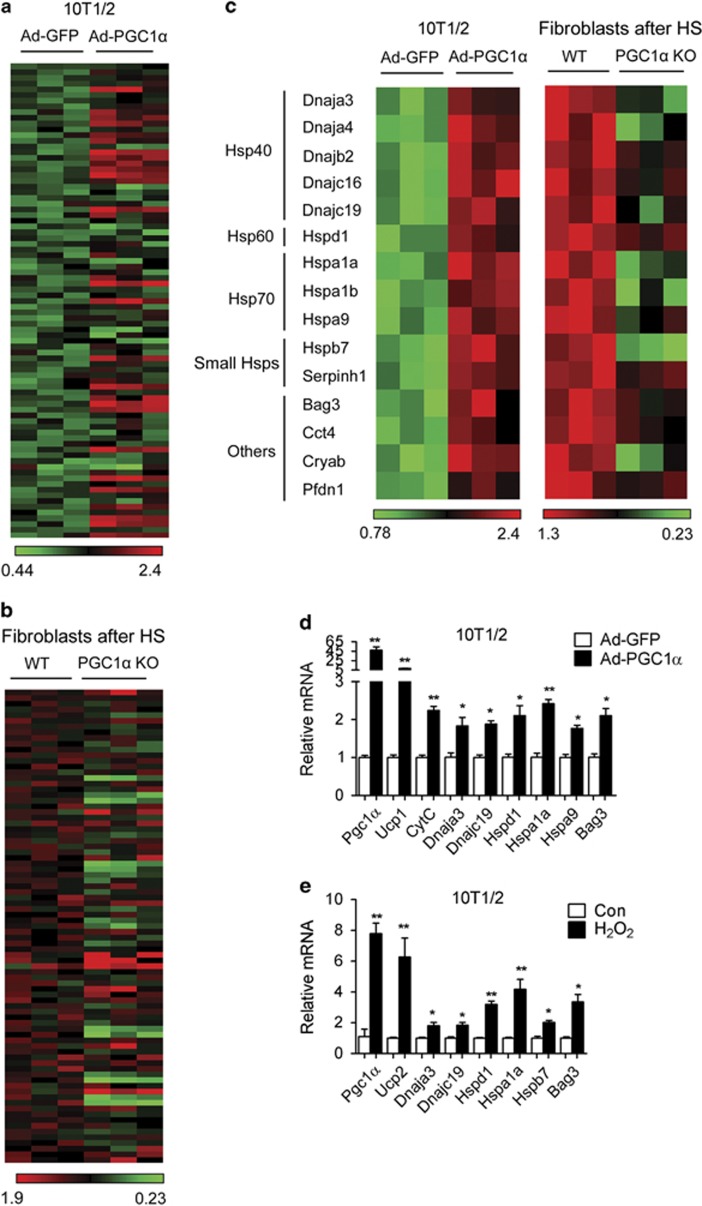
PGC1*α* is sufficient to induce the expression of HSPs i*n vitro*. (**a**–**c**) Heat map representation of 84 differentially regulated HSP genes (red, high; green, low) identified by PCR array analysis in 10T1/2 cells infected with control or PGC1*α* adenovirus for 3 days (**a**) and in WT and PGC1*α*-null fibroblasts exposed to heat shock for 1 h at 42 °C or at 37 °C (**b**) and of 15 common regulated HSP genes from two arrays (**c**). (**d**) mRNA levels of PGC1*α*, UCP1, CytC and HSPs in 10T1/2 cells infected with control or PGC1*α* adenovirus for 3 days. (**e**) mRNA levels of PGC1*α*, UCP2 and HSPs in 10T1/2 cells treated with H_2_O or 0.5 *μ*M hydrogen peroxide for 2 h and subsequently allowed to recover for 2 h. Error bars represent S.E.M. and data are presented as mean±S.E.M. *P<0.05, **P<0.01 compared with controls

Given our recent data demonstrating the existence of a transcriptional cooperation between PGC1*α* and HSF1 in the context of energy metabolism^21^ and the previously reported function of PGC1*α* in protection from stress,^18^ we hypothesized that PGC1*α* may have a role in cellular defense against heat shock through its cross talk with HSF1. To assess this, we first systematically analyzed the expressions of a number of genes known to be involved in heat shock responses in cells with gain- or loss-of-function of PGC1*α*. This analysis revealed that PGC1*α* ectopic expression in 10T1/2 cells significantly induced 32% of the genes commonly known to be involved in heat shock responses (Figure 1a and Supplementary Table 1). Conversely, PGC1*α* ablation in fibroblasts was associated with a reduction in the levels of 29% of these HSPs and chaperone genes when cells were exposed to heat shock for 1 h (Figure 1b and Supplementary Table 2). Cross comparison analysis of genes modulated both in cells with upregulation or downregulation of PGC1*α* revealed that 15 genes, including 5 HSP40s (Dnaja3, Dnaja4, Dnajb2, Dnajc16 and Dnajc19), 1 HSP60 (Hspd1), 3 HSP70s (Hspa1a, Hspa1b and Hspa9), 2 small HSPs family members (Hspb7/Hsp27 and Serpinh1/Hsp47) and 4 other chaperones (Bag3, Cct4, Cryab and Pfdn1) are possible targets of PGC1*α* given that their expression levels are dependent on PGC1*α* (Figure 1c). The increased levels of representative HSPs of different categories, such as Dnaj3, Dnajc19, HSPd1, HSPa1a, HSPa9 and Bag3, were further validated by real-time PCR in 10T1/2 cells overexpressing PGC1*α* (Figure 1d).

It has been previously demonstrated that PGC1*α* is a stress-induced protein, activated by a number of challenges, including ROS.^18^ We therefore next examined whether the mRNA levels of HSPs were regulated under the same conditions known to induce PGC1*α* and genes involved in ROS responses. As shown in Figure 1e, HSPs mRNAs were elevated following exposure to hydrogen peroxide, with UCP2 expression levels used as a positive control. These results demonstrate that PGC1*α* is both sufficient and necessary to induce the expression of HSPs *in vitro* in response to cellular stressors.

### PGC1*α* modulates HSP levels *in vivo*

**Figure 2:**
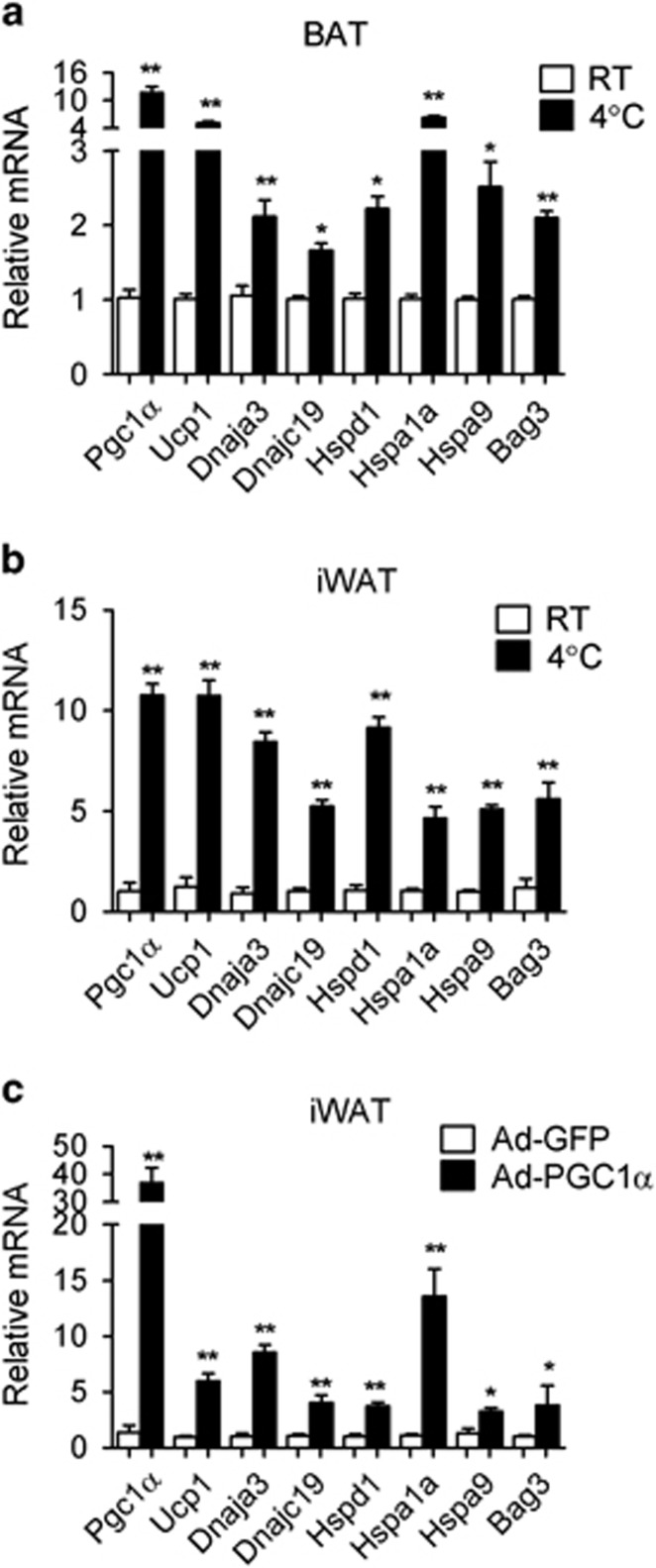
PGC1*α* modulates HSP expression *in vivo*. (**a** and **b**) mRNA levels of PGC1*α*, UCP1 and HSPs in BAT (**a**) and iWAT (**b**) of mice kept at room temperature or after cold exposure for 4 h. (**c**) mRNA levels of PGC1*α*, UCP1 and HSPs in iWAT injected with control or PGC1*α*-expressing adenovirus. Error bars represent S.E.M. and data are presented as mean±S.E.M. *P<0.05, **P<0.01 compared with controls. *n*=4 per group

We next examined whether PGC1*α* regulates HSPs also *in vivo*. Given that the expression of PGC1*α* is cold-inducible,^22^ we determined whether HSPs would be induced in mice exposed to 4 °C. As shown in Figures 2a and b, we observed an induction of HSPs in brown and inguinal fat in the same conditions that elicited an increase in the expression of PGC1*α* and UCP1 in these tissues, suggesting that the levels of these HSPs are regulated by hypothermia. Interestingly, the acute exposure to cold did not elevate the levels of HSPs in tissues such as heart and kidney in which PGC1*α* mRNAs are not regulated in response to low temperatures (Supplementary Figures 1a and b) nor damaged liver and kidney as indicated by the absence of apoptotic events in these two tissues (Supplementary Figure 1c).

To further examine whether gain of PGC1*α* function would be associated with the expression of HSP *in vivo*, we took advantage of adenoviral delivery of PGC1*α* in tissues by following the same technical procedures that we previously used to successfully achieve expression of specific genes in selected depots.^21, 23^ This analysis revealed that HSPs are induced in inguinal fat (iWAT) overexpressing PGC1*α* by adenoviral injection but not in control-injected iWAT (Figure 2c). Of note, these adenoviral injections did not alter the levels of PGC1*α* nor caused any morphological changes in organs such as brown adipose tissue (BAT), liver, kidney and heart (Supplementary Figures 1d and e).

### PGC1*α* is required for the HSF1-dependent induction of Hsp70 in hyperthermia

**Figure 3:**
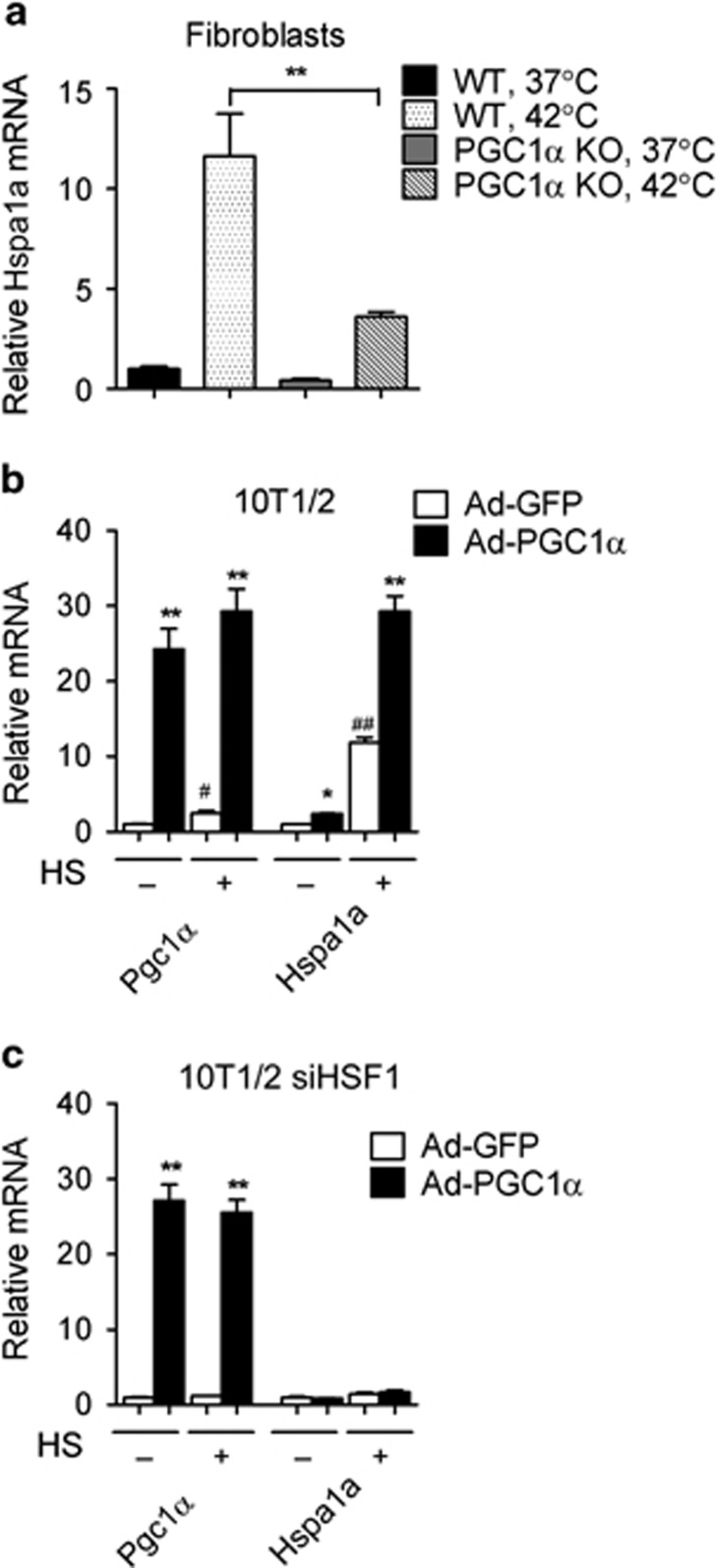
PGC1*α* is required for HSF1-dependent induction of the HSP70 family member Hspa1a. (**a**) mRNA levels of Hspa1a in WT and PGC1*α*-null fibroblasts exposed to heat shock (42 °C) for 1 h or left at 37 °C. (**b**) mRNA levels of Hspa1a in 10T1/2 cells infected with adenovirus expressing control or PGC1*α* and treated after 1 h of heat shock at 42 °C or at 37 °C. ^#^*P*<0.05, ^##^*P*<0.01, comparison of Ad-GFP group with or without heat shock (HS) treatment. (**c**) mRNA levels of Hspa1a in 10T1/2 cells with HSF1 knockdown, infected with either control or PGC1*α* adenovirus and subjected to heat shock at 42 °C for 1 h or left at 37 °C. Error bars represent S.E.M. and data are presented as mean±S.E.M. *P<0.05, **P<0.01 compared with controls

In order to investigate whether PGC1*α* is indispensable for the induction of HSPs in response to hyperthermia, we subjected WT and PGC1*α*-null cells to acute heat shock and analyzed the modulation of Hspa1a/Hsp70 levels given that this gene is among the most well-studied stress-inducible Hsp70 of all HSPs and its transcriptional regulation by HSF1 in response to heat shock is well documented. This analysis revealed that whereas Hspa1a/Hsp70 mRNA was induced in WT cells, as expected, its levels were blunted in PGC1*α*-null cells (Figure 3a and Supplementary Table 2). Conversely, PGC1*α* overexpression enhanced Hspa1a/Hsp70 levels after heat shock (Figure 3b). Given that the expression of HSPs is regulated by HSF1, we analyzed the effects of PGC1*α* overexpression in HSF1-deficient cells. As shown in Figure 3c and Supplementary Figure 2, ectopic expression of PGC1*α* was not sufficient to increase Hspa1a/Hsp70 levels in the absence of HSF1, suggesting that PGC1*α* requires the transcription factor HSF1 to modulate the expression of this member of the Hsp70 family.

### PGC1*α* and HSF1 physically interact and functionally cooperate to transcriptionally activate Hsp70

**Figure 4:**
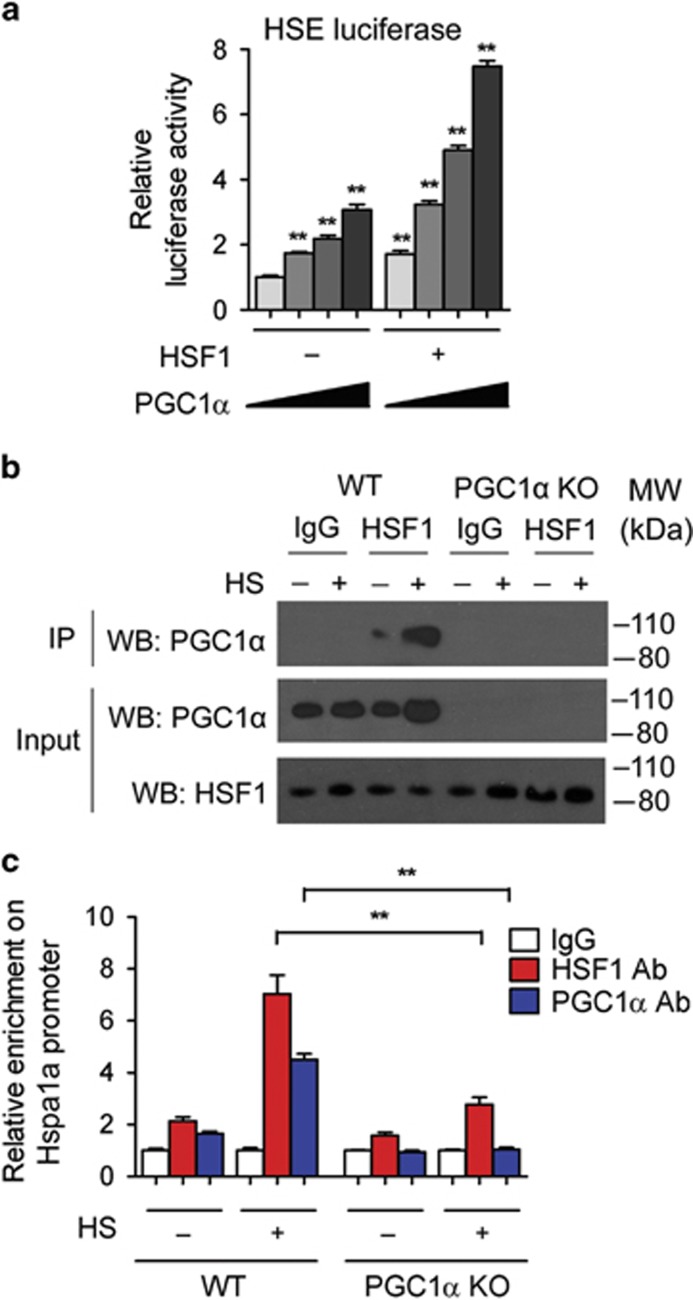
PGC1*α* and HSF1 physically interact and functionally cooperate on heat shock response elements. (**a**) Luciferase assay performed on a reporter containing a HSE in 10T1/2 cells co-transfected with PGC1*α*, HSF1 and vector controls. (**b**) Co-IP assay to assess the interaction between PGC1*α* and HSF1 in WT and PGC1*α*-null brown adipocytes exposed to heat shock at 42 °C (HS) for 1 h and recovered for an additional hour. (**c**) ChIP assay at the Hsp70 promoter after 1 h 42 °C heat shock and 1 h recovery in WT and PGC1*α*-null brown adipocytes to assess the binding of PGC1*α* and HSF1. Error bars represent S.E.M. and data are presented as mean±S.E.M. *P<0.05, **P<0.01 compared with controls

Given the requirements of PGC1*α* for the induction of the HSF1 target genes, we sought to determine the mechanisms through which PGC1*α* affects Hspa1a/Hsp70 mRNA levels. Transcriptional assays revealed that gain of PGC1*α* function led to the induction of luciferase driven by a HSE in a dose-dependent manner and that the coexpression of PGC1*α* and HSF1 further increased the activity of this reporter gene (Figure 4a), suggesting that PGC1*α* and HSF1 synergistically activate genes controlled by HSE. Furthermore, our analysis revealed that PGC1*α* and HSF1 physically interact and that their binding is enhanced during heat shock (Figure 4b). Through ChIP assay, we confirmed that PGC1*α* and HSF1 bind to the HSE element present at the Hsp70 promoter after heat shock. Conversely, we observed reduced binding of HSF1 to the HSE element present on the promoter of Hsp70 in PGC1*α*-null cells (Figure 4c and Supplementary Figure 3). Together, these data indicate that PGC1*α* interacts with HSF1 and cooperates in the transcriptional activation of Hsp70.

### PGC1*α*-deficient cells are sensitive to heat stress

**Figure 5:**
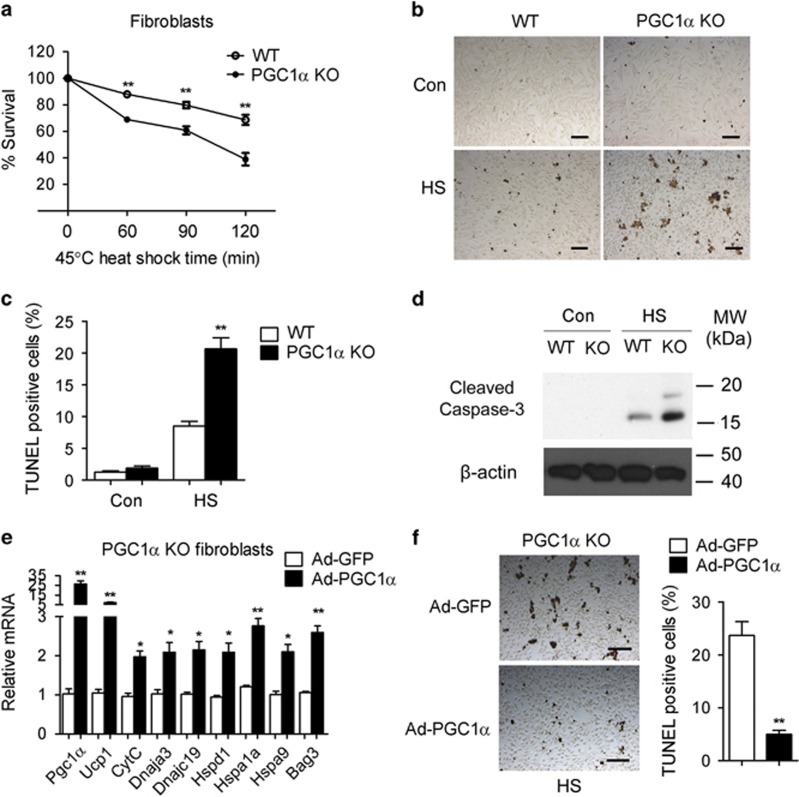
PGC1*α*-deficient cells are less resistant to heat stress. (**a**) Survival curve of WT and PGC1*α*-null fibroblasts after heat shock at 45 °C for the indicated time. (**b** and **c**) Representative images of TUNEL staining (**b**) and quantification of TUNEL-positive cells (**c**) in WT and PGC1*α*-null cells kept at 37 °C or exposed to heat shock at 43 °C for 1 h and recovered for 30 min at 37 °C. (**d**) Protein levels of cleaved Caspase-3 in WT and PGC1*α*-null cells after exposure to heat shock at 43 °C for 1 h and recovered for 30 min at 37 °C or in control cells kept at 37 °C. (**e**) mRNA levels of HSPs in PGC1*α*-null cells expressing control or complemented with PGC1*α*. (**f**) Representative images of TUNEL staining and quantification of TUNEL-positive cells in PGC1*α*-null cells either with control or re-expressing PGC1*α* after heat shock at 43 °C for 1 h and recovery for 30 min at 37 °C. Scale bar represents 100 *μ*m. Error bars represent S.E.M. and data are presented as mean±S.E.M. *P<0.05, **P<0.01 compared with controls

Given our results indicating impaired induction of HSPs after hyperthermic stress in cells lacking PGC1*α* (Supplementary Table 2 and Figure 3a), we tested whether PGC1*α* is necessary for cellular protection from hyperthermia. As shown in Figures 5a–c, when PGC1*α*-null cells were exposed to heat shock, they showed reduced viability (Figure 5a) and increased apoptosis in comparison with WT cells (Figures 5b and c). Consistently, we observed increased cleavage of caspase-3 in PGC1*α*-null cells exposed to heat shock (Figure 5d). Complementation studies demonstrated that restoration of PGC1*α* levels in PGC1*α*-null cells by adenovirus was associated with elevation of HSP levels (Figure 5e) and increased resistance to apoptosis in response to heat stress (Figure 5f). These data indicate that PGC1*α* has critical and unexpected roles in regulating cellular responses to hyperthermia.

### PGC1*α* is required for heat resistance in response to thermal challenge in mice

**Figure 6:**
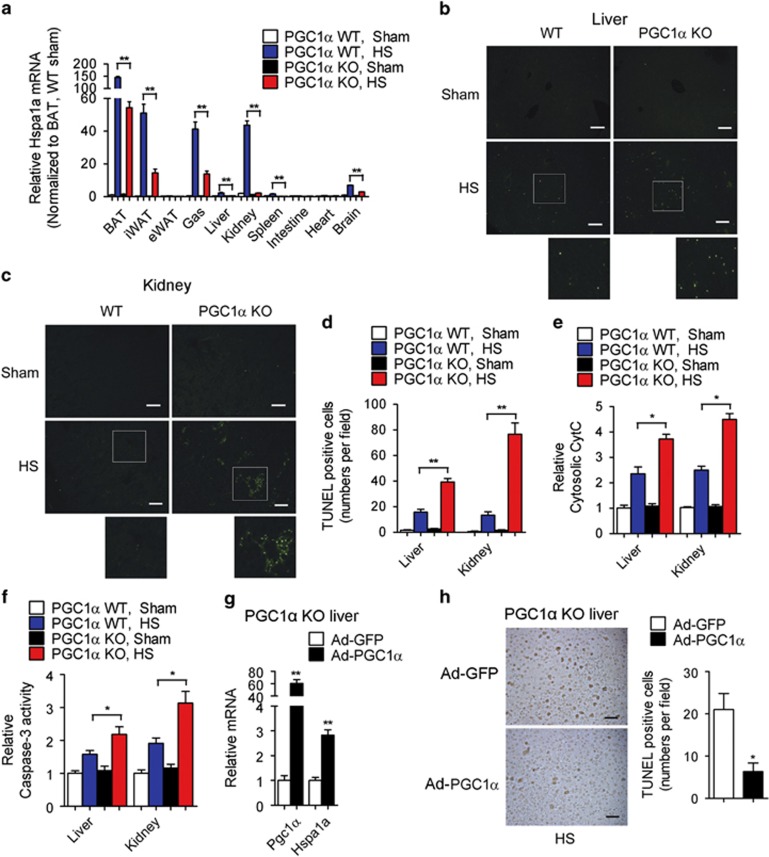
PGC1*α*-null mice exhibit impaired responses to thermal challenges. (**a**) mRNA levels of Hspa1a in tissues of WT and PGC1*α*-null mice exposed to heat shock or kept at room temperature (RT). (**b** and **c**) Representative images of TUNEL staining in liver and kidney from WT and PGC1*α*-null mice after heat shock or at RT. Images from heat shock groups were magnified digitally (below) to show details of the staining. Scale bar represents 100 *μ*m. (**d**) Quantification of numbers of TUNEL-positive cells per field in liver and kidney sections from WT and PGC1*α*-null mice exposed to heat shock or kept at RT. (**e** and **f**) Relative levels of released cytochrome c in cytosol and Caspase-3 activity in liver and kidney from WT and PGC1*α*-null mice exposed to heat shock or kept at RT. (**g**) mRNA level of Pgc1*α* and Hspa1a in livers from PGC1*α*-null mice either infected with control or PGC1*α* through tail vain injection of adenovirus. *n*=3 per group. (**h**) Representative images of TUNEL staining and quantification of TUNEL-positive cells in livers from PGC1*α*-null mice either with control or re-expressing PGC1*α* after heat shock. Scale bar represents 200 *μ*m. *n*=3 per group. Error bars represent S.E.M. and data are presented as mean±S.E.M. *P<0.05, **P<0.01 compared with controls. *n*=5 per group

In order to characterize the *in vivo* significance of PGC1*α* function during hyperthermic stress, we exposed WT and PGC1*α*-null mice to sub-lethal temperatures. Analysis of organs including BAT, iWAT, gastrocnemius, kidney, liver, spleen and brain revealed an elevation of Hspa1a/Hsp70 levels after heat shock in WT mice compared with mice lacking PGC1*α*, suggesting that PGC1*α* is required for Hsp70 induction after hyperthermia *in vivo* (Figure 6a). Furthermore, we observed an increase in PGC1*α* levels in parallel to those of Hsp70 in response to heat, supporting the possibility that HSF1 induces PGC1*α* and that requires it for HSP induction (Supplementary Figure 4a). Given the evidence that PGC1*α* can protect against apoptosis induced by hyperthermia *in vitro* (Figure 5) and that liver and kidney are the primary loci affected during heat stroke in humans,^24^ we analyzed WT and PGC1*α* KO mice and showed increased apoptosis, increased cytosolic cytochrome c (CytC) release from mitochondria and elevated caspase-3 activity in these two organs in PGC1*α*-null mice after heat shock (Figures 6b–f). In addition, restoration of PGC1*α* levels in liver of PGC1*α*-null mice by tail vein adenoviral injections was associated with increased Hspa1a mRNA levels (Figure 6g) and enhanced resistance to apoptosis after heat shock (Figure 6h). These results suggest that absence of PGC1*α* is associated with reduced levels of HSPs and impaired responses to thermal challenges.

## Discussion

The analyses performed here demonstrate that PGC1*α* can cooperate with HSF1 in the induction of a number of HSPs in a variety of tissues and cells in response to different stimuli, such as thermal and oxidative stress. Furthermore, loss-of-function studies showed that PGC1*α* is required for HSF1 activation of HSPs and protection from hyperthermia. This cytoprotective role of PGC1*α* exerted through the activation of HSPs is consistent with the previously characterized function of this coactivator in cell defense in response to a variety of insults, as reported in the context of transcriptional activation of detoxifying genes in cells exposed to ROS^18^ and in the unfolded protein response regulated in cooperation with ATF6.^25^ It is plausible that PGC1*α* may complex with other transcription factors in addition to HSF1 to induce stress-induced proteins; therefore, future studies involving mass spectrometry and ChIP-seq analysis will be instructive in defining which tissue-selective transcriptional factors cooperate with PGC1*α* to coordinate organ-selective responses to distinct stressors.

Our analysis revealed for the first time that PGC1*α* is necessary for the induction of Hspa1a/Hsp70/Hsp72. Given the evidence provided by previous studies demonstrating that this HSP is induced during cold exposure^26^ and via gain- and loss-of-function analyses showing its requirements for cold resistance *in vivo*,^27^ it can be postulated that Hspa1a/Hsp70/Hsp72 may be an essential target of PGC1*α* for the expletion of its thermogenic function. In addition to its role in protecting from oxidative stress, this Hsp has also been shown to be involved in the control of insulin resistance through its negative effects on JNK phosporylation.^28^ Given the previously recognized role of PGC1*α* in energy expenditure and insulin sensitivity, it is conceivable that Hspa1a/Hsp70/Hsp72 may mediate other metabolic beneficial effects elicited by PGC1*α*, in addition to its function in cytoprotection in response to hyperthermic stresses.

It has been previously demonstrated that PGC1*α* is involved in organelle remodeling and biogenesis.^16, 17^ The data reported here suggest that mitochondrial HSP proteins such as Hspd1/HSP60 and Hspa9/mtHSP70 devoted to organelle protection from proteotoxic stress are regulated by PGC1*α*. Consistently with our findings, Rera *et al.*^29^ demonstrated that *drosophila* overexpressing PGC1*α* have increased HSP60 levels in both whole larvae and adult thoraxes and increased mitochondrial activity. Given that these mitochondrial HSPs have been postulated to be under the transcriptional control of HSF1,^30^ our results provide novel insights into the broader function of PGC1*α* and its transcriptional partners in the regulation of mitochondrial biology.

Genetic mouse models have demonstrated that ablation of HSPs such as HSP70 and HSP60 can cause neurological defects including cerebral ischemia, Huntington's disease and motor neuron disorders.^31, 32, 33^ Given that it has been previously shown that ablation of PGC1*α in vivo* is associated with brain abnormalities,^18, 19^ it is possible that part of the PGC1*α*-null mice phenotype may be due to decreased levels of HSPs in this organ. Taken together, our study demonstrates for the first time that PGC1*α* regulates HSPs and protects against hyperthermic stress in cooperation with HSF1, indicating a critical novel mechanism of cellular protection exerted by PGC1*α* and suggesting a broader role of PGC1*α* in cellular defense in response to a variety of insults.

## Materials and Methods

### Cell culture

10T1/2 cells were maintained in DMEM supplemented with 10% fetal bovine serum (FBS). For proteotoxic stress treatment, 10T1/2 cells were treated with hydrogen peroxide (Sigma, 216763, St. Louis, MO, USA). Immortalized WT and PGC1*α*-null mouse fibroblasts, a generous gift of Bruce Spiegelman (Harvard Medical School, Boston, MA, USA), were maintained in DMEM supplemented with 10% FBS, 20 nM insulin and 1 nM T3 (maintenance medium). Differentiation was induced by treating confluent cells with 0.5 mM isobutylmethylxanthine, 125 *μ*M indomethacin and 0.5 *μ*M dexamethasone for 2 days. For adenoviral experiments, 10T1/2 cells, WT or PGC1*α*-null fibroblasts were infected with 600 MOI of adenovirus (control and PGC1*α* adenoviruses were constructed, amplified and purified by Vector Biolabs, Malvern, PA, USA) for 2 h in 0.5% FBS medium and subsequently cultured in 10% FBS medium. Ten percent FBS-containing medium was changed after 24 h and gene expression was determined 72 h after adenovirus infection, in cells exposed to heat shock or 37 °C as control in a water bath placed in the incubator. For viability measurements, cells were stained with trypan blue after exposure to 45 °C for the indicated time and the percentage of live cells was quantified using a haemocytometer.

### Plasmids and reagents

The HSE luciferase reporter vector was purchased from Affymetrix Panomics (LR0038, Affymetrix, Santa Clara, CA, USA). The PGC1*α*- and HSF1-expressing plasmids were kind gifts of Bruce Spiegelman (Harvard Medical School) and Carl Wu (National Cancer Institute), respectively. For luciferase assay, 10T1/2 cells plated in 24 wells were transfected with 0.1 *μ*g of HSE luciferase reporter, 0, 25, 50 or 100 ng of PGC1*α* plasmids in the presence or absence of 10 ng of HSF1 using a Nucleofector 96-well system (Amaxa, Lonza, Basel, Switzerland), according to the manufacturer's instructions. Control plasmid was used to even the total amount of DNA transfected in each well. Luciferase activity was assayed 48 h after transfection, according to protocols provided by the manufacturer (Promega, Fitchburg, WI, USA) using VICTOR3V (PerkinElmer, Waltham, MA, USA).

### RNA analysis and PCR arrays

Total RNA was extracted from cultured cells or tissues with TRIzol (Invitrogen, Carlsbad, CA, USA) and 1 *μ*g total RNA was retrotranscribed into cDNA with First Strand cDNA Synthesis Kit (Roche, Basel, Switzerland), according to the manufacturers' instructions. Quantitative real-time PCR was performed using with the ABI PRISM 7900HT sequence detection system (Applied Biosystems, Thermo Scientific, Waltham, MA, USA) using SYBR green (Roche). Gene expression levels were determined by the delta delta Ct method, normalized to 36B4 expression levels. The sequence of primers used for real-time PCR is reported in Supplementary Table 3. One microgram of total RNA from 10T1/2 cells infected with Ad-control or Ad-PGC1*α* or WT and PGC1*α* null fibroblasts after 1 h heat shock was retrotranscribed into cDNA and used to perform Mouse Heat Shock Proteins & Chaperones PCR arrays (PAMM-076Z, Qiagen, Hilden, Germany) according to the manufacturer's instructions.

### Animal experiments

All animal experiments were performed according to guidelines of the National Institute of Diabetes and Digestive and Kidney Diseases' Animal Care and Use Committee. PGC1*α*-heterozygous mice were a generous gift from BM Spiegelman (Harvard Medical School). To test gene expressions and apoptotic events after cold exposure, mice were individually caged and exposed to 4 °C or maintained at room temperature with free access to water for 3 or 4 h before killing them. For thermal challenge, 2-month-old male PGC1*α*-null mice and WT littermates, or PGC1*α*-null mice infected with control or Ad-PGC1*α* through tail vein injections, were anesthetized with isoflurane gas, immobilized and covered with a blanket and placed under a 250 W infrared lamp. Core rectal temperatures were monitored with a rectal thermometer (TH5, Braintree Scientific, Braintree, MA, USA) and raised to 41±0.5 °C and kept at 41 °C for 15 min. After thermal challenge, mice were slowly returned to isothermal conditions over a warm blanket. Mice were killed and dissected 6 h after thermal challenge. The mice of the control group were also anesthetized with isoflurane gas, immobilized and covered with a blanket and monitored with a rectal thermometer in parallel, but maintained at room temperature for the same amount of time as the heat shock group.

### Adenoviral delivery in inguinal fat and liver

Adenoviruses expressing control (CMV-GFP) and PGC1*α* (mPGC1a-CMV-GFP) were constructed, amplified and purified by Vector Biolabs. Each of the adenoviruses (5 × 10^9^ pfu) diluted in 50 *μ*l saline were injected into the inguinal fat pads of mice or 2 × 10^9^ pfu adenovirus through tail vein injection. Mice were killed at the forth day after viral delivery and tissues were dissected for gene analysis or for further heat shock treatment.

### TUNEL assay and H&E staining

WT, PGC1*α*-null cells and null cells with PGC1*α* levels reconstituted were maintained in the incubator in a water bath at 37 °C or 43 °C for 1 h then exposed to a recovery time of 30 min at 37 °C. Cells were then fixed with fresh 4% paraformaldehyde and permeabilized with 0.1% Triton X-100. Kidneys and livers from WT and PGC1*α* KO exposed to cold for 3 h and liver of PGC1*α* KO mice infected with control or PGC1*α* were dissected and tissues were fixed in 4% paraformaldehyde. Paraffin-embedded tissues were cut into sections of 5 *-μ*m thickness. TUNEL assay (terminal deoxynucleotidyl transferase-mediated dUTP-biotin nick end labeling) was performed to assess DNA fragmentation according to instructions provided by the manufacturer (Roche, 11 684 817 910). Cells or tissue slides were analyzed using a microscope (Olympus, Tokyo, Japan) and images captured by a digital camera (Olympus) with 100- or 200-fold magnification. Positive control was performed on a liver section treated with Dnase I prior to labeling procedure. To quantify TUNEL-positive cells, four individual wells or tissue slides from mice of each genotype were assayed. Ten fields were then randomly selected per well or slide. TUNEL-positive cells were counted on each field and averaged (percentage of positive cells per field was shown for cells and numbers of positive cells per field were shown for tissues). For toxicity analysis, BAT, liver, kidney and heart of mice injected with control or Ad-PGC1*α* in iWAT were dissected and fixed in 4% paraformaldehyde and paraffin-embedded tissues were cut into section of 5*-μ*m thickness for H&E staining following standard procedures.^21, 23^

### Caspase-3 activity and cytosol cytochrome c (CytC) level measurement

Measurement of liver and kidney caspase-3 activity was performed using the Caspase-3 Colorimetric Assay Kit (Abcam, ab39401, Cambridge, UK) following the manufacturer's instructions. Livers and kidneys were cut into small pieces, washed three times with supplied cell lysis buffer and homogenized with a tissue grinder. Liver and kidney homogenates were centrifuged for 10 min at 12 000 r.p.m. and supernatants were used for colorimetric determination at 405 nm. The OD values were normalized to the protein levels present in the tissues. The homogenates were also used to isolate cytosolic fraction with Standard Cell Fractionation Kit (Abcam, ab109719) and cytosolic CytC levels were determined by Cytochrome c Profiling ELISA Kit (Abcam, ab110172) and normalized to protein levels present in the tissues.

### Co-immunoprecipitation assays and western blots

Lysates from differentiated WT and PGC1*α*-null cells were incubated with anti-HSF1 antibody (Abgent, San Diego, CA, USA, AJ1374a) and rabbit IgG (Santa Cruz, Dallas, TX, USA, sc-2027) and performed on Catch and Release v2.0 reversible immunoprecipitation system following the manufacturer's instructions (Millipore, Billerica, MA, USA). The immune complexes were eluted and subjected to SDS-PAGE. For immunoblot detection, we used anti-PGC1*α* (Santa Cruz, sc-13067) and anti-HSF1 (Abgent AJ1374a antibodies). The secondary goat anti-rabbit antibody was purchased from Santa Cruz (sc-2030). For western blot, proteins were extracted from cells using RIPA buffer, consisting of 20 mM Tris, 150 mM NaCl, 1% Triton X-100 and protease inhibitors (Roche) and separated in 4–20% or 10% Bis-Tris Gel (ThermoFisher, Rockville, MD, USA), transferred onto a PVDF membrane (Pierce, ThermoFisher) and incubated with primary antibodies including anti-Cleaved Caspase-3 (Cell Signaling, 9661, Danvers, MA, USA) and anti-*β*-actin (Sigma, A5316), overnight at 4 °C. Novex Sharp Pre-stained Protein Standard (ThermoFisher) was used as protein molecular size marker.

### Chromatin immunoprecipitation assays

ChIP analyses of WT and PGC1*α*-null cells were performed using a ChIP assay kit (Millipore), following the manufacturer's recommendation and as previously described.^21^ Sonicated cell lysates were incubated with anti-IgG (Santa Cruz, sc-2027), anti-PGC1*α* (Santa Cruz, sc-13067) or anti-HSF1 (Abgent, AJ1374a) antibodies for protein-DNA binding detection. The sequences of the primers used to assess the binding of HSF1 on the mouse Hsp70 promoter (-272 to +47) were the following: F: 5'- CAACAGTGTCACTAGTAGCACC-3' and R: 5'- CTCTGGATGGAACCA GATTTGG-3' as previously described^34^ and on *β*-globin: F: 5'-AAGCCTGATTCCGTAGAGCCACAC-3' and R: 5'-CCCACAGGCAAGAGACAGCAGC-3'AAGCCTGATTCCGTAGAGCCACAC.^21^

### Statistical analysis

Comparisons between groups were performed using *t*-test (GraphPad Software). Differences were considered significant with *P*<0.05. Error bars represent S.E.M. and results are shown as means± S.E.M.

## Abbreviations

HSP: heat shock protein
ATP: adenosine triphosphate
ADP: adenosine diphosphate
HSE: heat shock element
ROS: reactive oxygen species
MPTP: (1-methyl-4-phenyl-1,2,3,6-tetrahydropyridine)
S.E.M.: standard error of the mean
DMEM: Dulbecco's modified Eagle's medium
FBS: fetal bovine serum
GFP: green fluorescent protein
