# Supplementary material for: The transcriptional coactivator PGC1α protects against hyperthermic stress via cooperation with the heat shock factor HSF1
Source: Cell Death Dis. 2016 Feb 18;7(2):e2102–. doi: 10.1038/cddis.2016.22 (PMC5399192; doi:10.1038/cddis.2016.22)
Supplement: Supplementary Information [file cddis201622x1.doc]

**SUPPLEMENTARY FIGURE LEGENDS**

**Supplementary Figure 1** PGC1α and heat shock proteins in heart and kidney of mice exposed to RT or cold, apoptotic analysis in liver and kidney after cold exposure and PGC1α mRNA levels and HE staining of multiple organs in mice with adenoviral injection in iWAT.

(a and b) mRNA levels of PGC1α, CytC and HSPs in heart (a) and kidney (b) in mice exposed to RT or cold for 4 hours.

(c) TUNEL staining of apoptotic cells in liver and kidney of mice after 3 hours of cold exposure at 4°C. Positive control was performed on a liver section treated with DNaseI prior to labeling procedure. *n*=3 per group and scale bar represents 200μm.

(d and e) Relative PGC1α mRNA levels (d) and HE staining (e) in BAT, liver, kidney and heart of mice adenoviral injection of GFP or PGC1α in iWAT.

Error bars represent SEM and data is presented as mean±SEM. *n*=4 per group.

**Supplementary Figure 2** HSF1 mRNA levels in 10T1/2 cells expressing control or siHSF1.

mRNA levels of HSF1 in 10T1/2 cells with knockdown of HSF1, infected with either control or PGC1α adenovirus, after exposure for 1 hour to heat shock at 42°C or maintained at RT.

Error bars represent SEM and data is presented as mean±SEM. **P<0.01 compared to controls.

**Supplementary Figure 3** ChIP assay at the β-globin promoter using PGC1α and HSF1 antibodies in WT and PGC1α-null brown adipocytes kept in 37°C or after heat shock at 42°C for 1 hour and after recovery for 1 hour. Error bars represent SEM and data is presented as mean±SEM.

**Supplementary Figure 4** PGC1α mRNA levels in multiple tissues from WT and PGC1α-null mice exposed to heat shock (HS) or at room temperature.

Error bars represent SEM and data is presented as mean±SEM. *P<0.05; **P<0.01 compared to controls. *n*=5 per group.
