# Supplementary figures and images for: The transcriptional coactivator PGC1α protects against hyperthermic stress via cooperation with the heat shock factor HSF1
Source: Cell Death Dis. 2016 Feb 18;7(2):e2102–. doi: 10.1038/cddis.2016.22 (PMC5399192; doi:10.1038/cddis.2016.22)

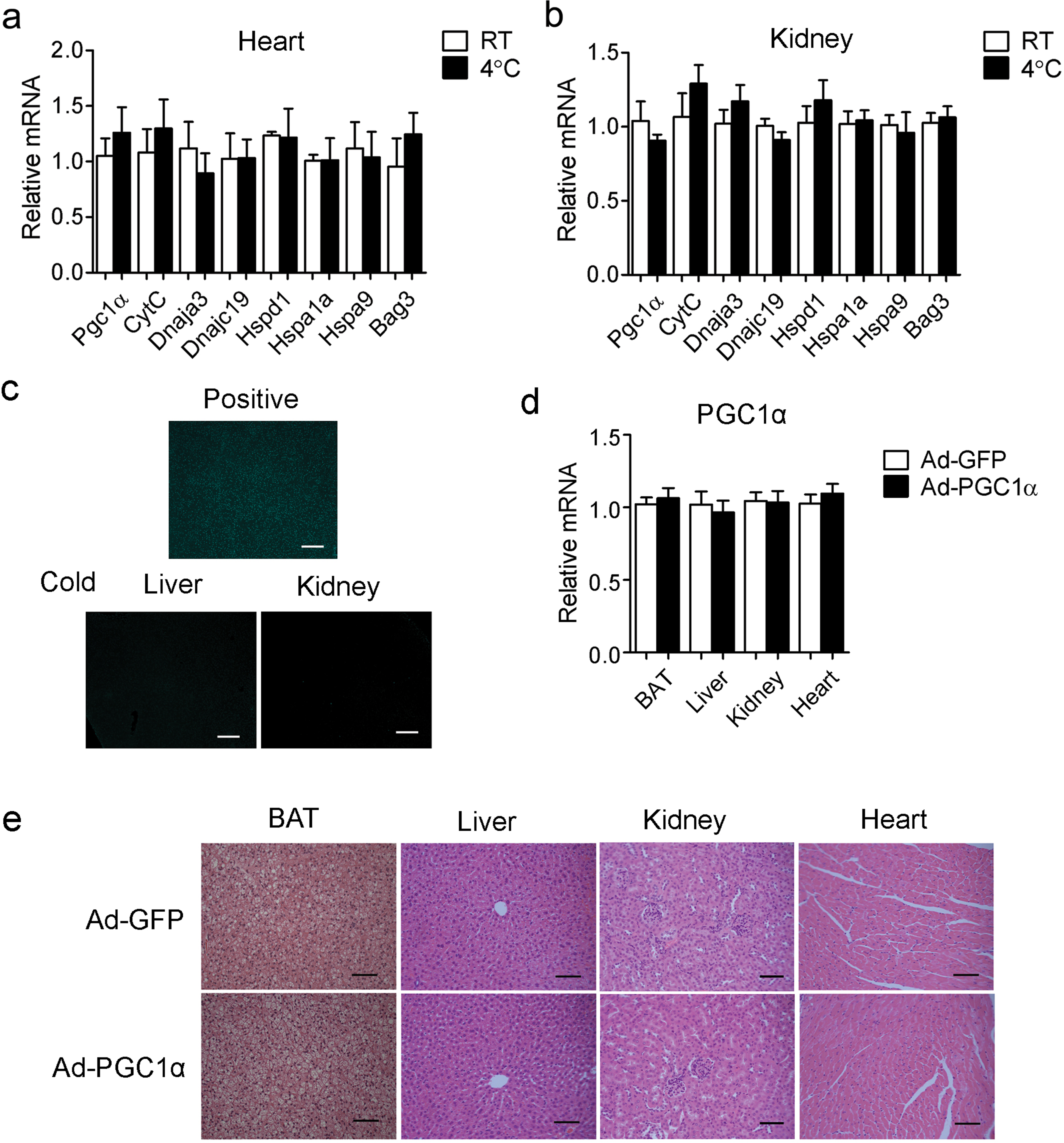

Supplement: Supplementary Figure 1 [file cddis201622x2.tif]

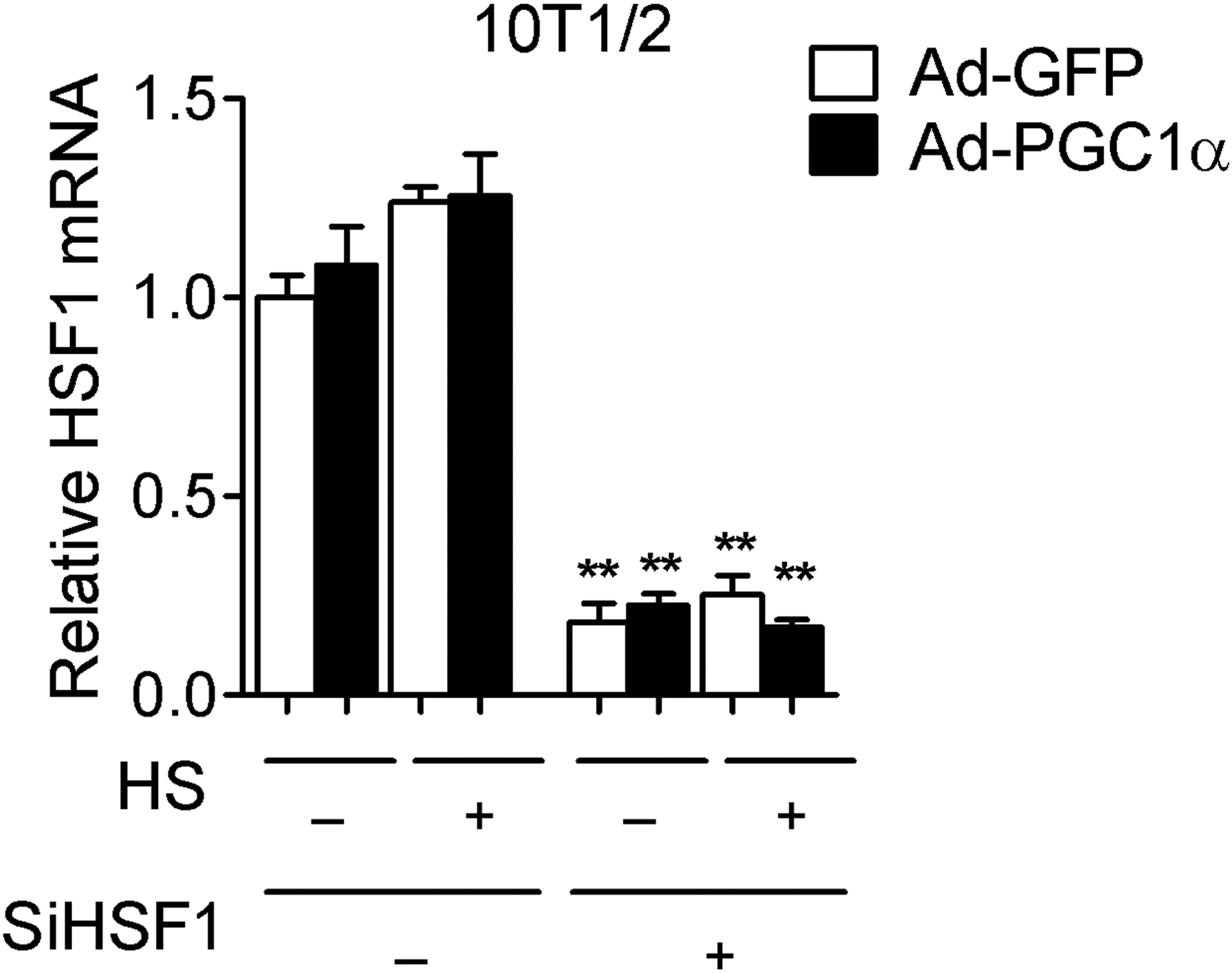

Supplement: Supplementary Figure 2 [file cddis201622x3.tif]

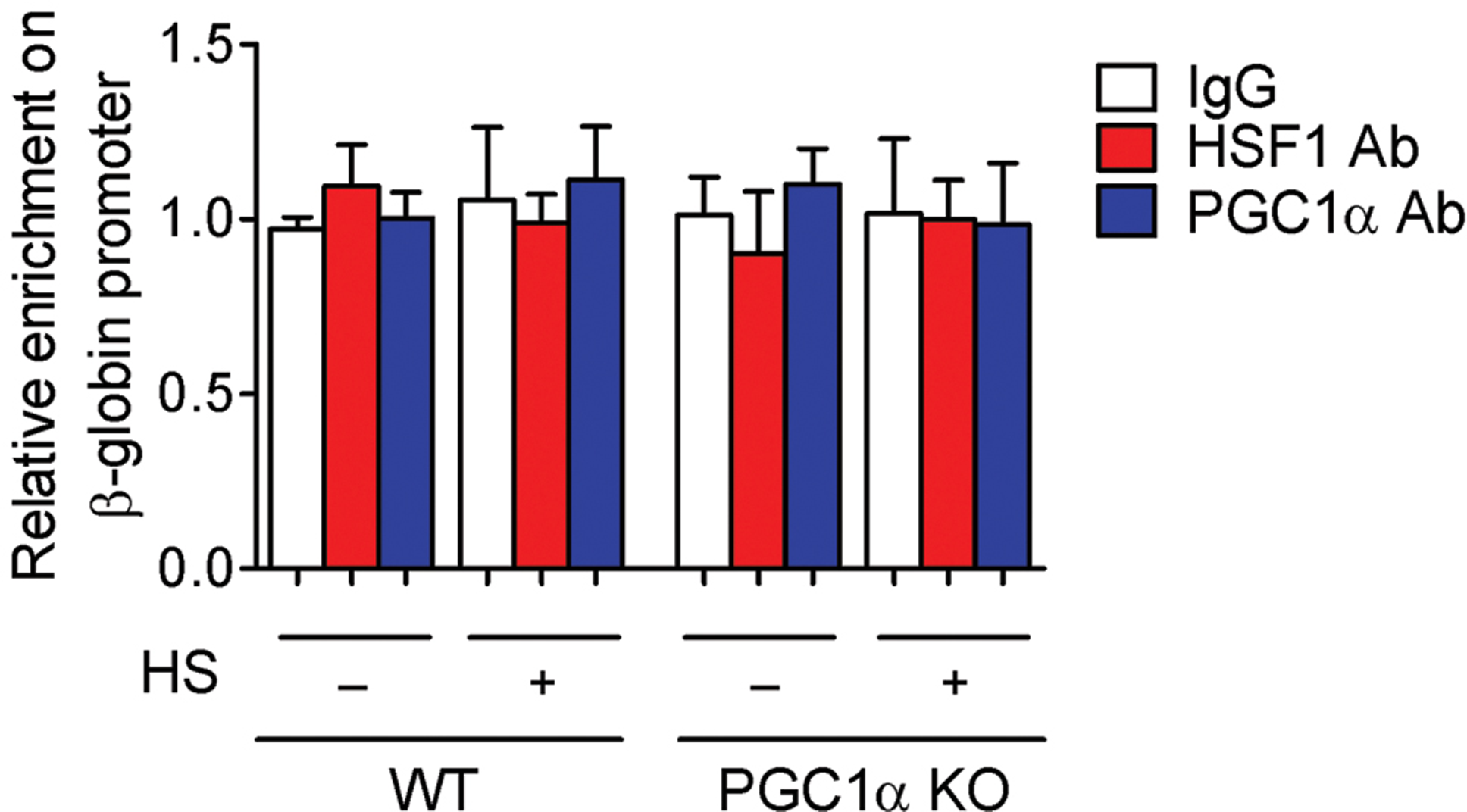

Supplement: Supplementary Figure 3 [file cddis201622x4.tif]

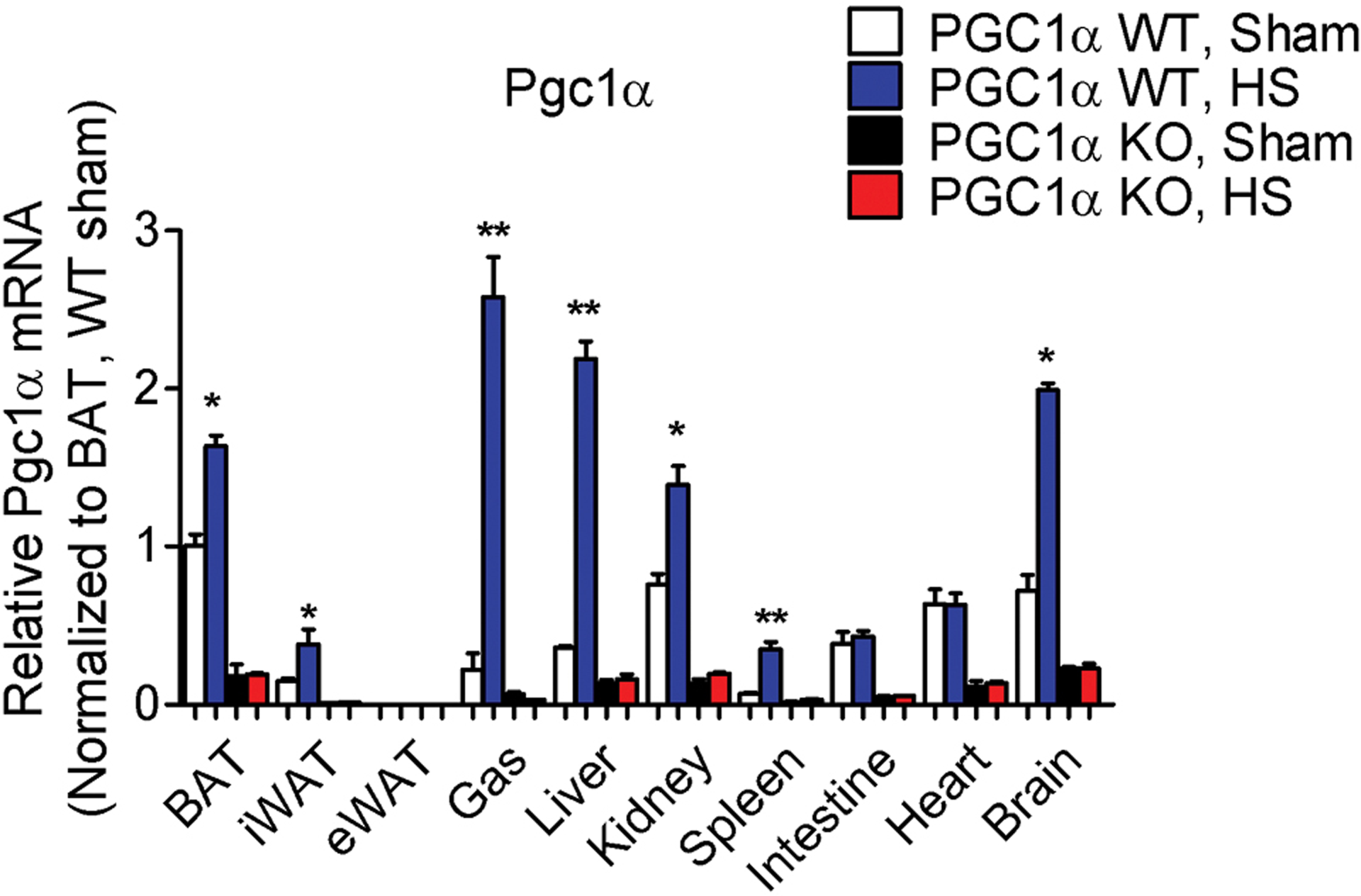

Supplement: Supplementary Figure 4 [file cddis201622x5.tif]
