# Supplementary material for: The transcriptional coactivator PGC1α protects against hyperthermic stress via cooperation with the heat shock factor HSF1
Source: Cell Death Dis. 2016 Feb 18;7(2):e2102–. doi: 10.1038/cddis.2016.22 (PMC5399192; doi:10.1038/cddis.2016.22)
Supplement: Supplementary Table 1 [file cddis201622x6.doc]

**Supplementary Table 1** Heat Shock Proteins & Chaperones array results for control and PGC1α overexpression in 10T1/2 cells.

| Gene | Fold  (OE vs Con) | P | Gene | Fold  (OE vs Con) | P | Gene | Fold  (OE vs Con) | P | Gene | Fold  (OE vs Con) | P |
| --- | --- | --- | --- | --- | --- | --- | --- | --- | --- | --- | --- |
| Adck3 | 1.09 | 0.095 | Dnajb1 | 1.52 | 0.047* | **Dnajc19** | **1.70** | **0.046*** | Hspa2 | 1.16 | 0.162 |
| Atf6 | 1.32 | 0.077 | Dnajb11 | 0.82 | 0.098 | Dnajc21 | 1.14 | 0.076 | Hspa4 | 1.21 | 0.113 |
| Bag1 | 1.13 | 0.119 | Dnajb12 | 1.11 | 0.155 | Dnajc3 | 1.10 | 0.141 | Hspa4l | 0.96 | 0.646 |
| Bag2 | 1.21 | 0.191 | Dnajab13 | 0.94 | 0.565 | Dnajc4 | 1.20 | 0.151 | Hspa5 | 1.14 | 0.096 |
| **Bag3** | **1.72** | **0.031*** | Dnajb14 | 1.14 | 0.217 | Dnajc5 | 1.13 | 0.202 | Hspa8 | 1.21 | 0.121 |
| Bag4 | 1.13 | 0.173 | **Dnajb2** | **1.74** | **0.042*** | Dnajc5b | 0.75 | 0.093 | **Hspa9** | **1.72** | **0.026*** |
| Bag5 | 1.12 | 0.375 | Dnajb5 | 1.35 | 0.599 | Dnajc5g | 0.76 | 0.082 | Hspb1 | 1.00 | 0.973 |
| Ccs | 1.41 | 0.147 | Dnajb6 | 1.13 | 0.189 | Dnajc6 | 0.86 | 0.356 | Hspb2 | 0.91 | 0.465 |
| Cct2 | 1.58 | 0.033* | Dnajb7 | 0.87 | 0.090 | Dnajc7 | 1.13 | 0.145 | Hspb3 | 0.67 | 0.071 |
| Cct3 | 1.60 | 0.029* | Dnajb8 | 0.72 | 0.148 | Dnajc8 | 1.09 | 0.238 | Hspb6 | 1.23 | 0.232 |
| **Cct4** | **1.64** | **0.019*** | Dnajb9 | 1.09 | 0.147 | Dnajc9 | 1.54 | 0.028* | **Hspb7** | **1.76** | **0.044*** |
| Cct5 | 1.14 | 0.182 | Dnajc1 | 1.13 | 0.166 | Hsf1 | 1.18 | 0.112 | Hspb8 | 1.31 | 0.062 |
| Cct6a | 1.45 | 0.041* | Dnajc10 | 1.19 | 0.176 | Hsf2 | 1.28 | 0.230 | **Hspd1** | **1.66** | **0.046*** |
| Cct6b | 0.95 | 0.763 | Dnajc11 | 1.24 | 0.134 | Hsf4 | 1.17 | 0.489 | Hspe1 | 1.33 | 0.074 |
| Cct7 | 1.50 | 0.032* | Dnajc12 | 0.88 | 0.306 | Hsp90aa1 | 1.32 | 0.113 | Hsph1 | 1.07 | 0.393 |
| Cryaa | 0.80 | 0.088 | Dnajc13 | 1.36 | 0.108 | Hsp90ab1 | 1.63 | 0.035* | **Pfdn1** | **1.58** | **0.041*** |
| **Cryab** | **1.85** | **0.005**** | Dnajc14 | 1.06 | 0.239 | Hsp90b1 | 1.10 | 0.174 | Pfdn2 | 1.24 | 0.101 |
| Dnaja1 | 1.61 | 0.002** | Dnajc15 | 1.34 | 0.087 | Hspa14 | 1.68 | 0.025* | **Serpinh1** | **1.75** | **0.025*** |
| Dnaja2 | 1.57 | 0.043* | **Dnajc16** | **1.93** | **0.001**** | **Hspa1a** | **1.95** | **0.014*** | Sil1 | 1.76 | 0.027* |
| **Dnaja3** | **1.76** | **0.034*** | Dnajc17 | 1.09 | 0.503 | **Hspa1b** | **1.77** | **0.002**** | Tcp1 | 1.13 | 0.214 |
| **Dnaja4** | **1.82** | **0.019*** | Dnajc18 | 1.17 | 0.398 | Hspa1l | 1.03 | 0.617 | Tor1a | 1.60 | 0.023* |

mRNA levels of HSPs in 10T1/2 cells infected with control or PGC1α adenovirus for 3 days. OE, PGC1α overexpression; CON, Control; *, P<0.05 and **, P<0.01. Genes with bold fonts highlight common genes that up-regulated with PGC1α overexpression (Supplementary Table 1) and down-regulated in PGC1α KO cells after heat shock (Supplementary Table 2).
