# Supplementary material for: The transcriptional coactivator PGC1α protects against hyperthermic stress via cooperation with the heat shock factor HSF1
Source: Cell Death Dis. 2016 Feb 18;7(2):e2102–. doi: 10.1038/cddis.2016.22 (PMC5399192; doi:10.1038/cddis.2016.22)
Supplement: Supplementary Table 2 [file cddis201622x7.doc]

**Supplementary Table 2** Heat Shock Proteins & Chaperones array results for WT and PGC1α KO cells after 42°C heat shock for 1 hour.

| Gene | Fold  (KO vs WT) | P | Gene | Fold  (KO vs WT) | P | Gene | Fold  (KO vs WT) | P | Gene | Fold  (KO vs WT) | P |
| --- | --- | --- | --- | --- | --- | --- | --- | --- | --- | --- | --- |
| Adck3 | 1.36 | 0.121 | Dnajb1 | 0.91 | 0.217 | **Dnajc19** | **0.69** | **0.030*** | Hspa2 | 1.32 | 0.122 |
| Atf6 | 1.04 | 0.474 | Dnajb11 | 1.76 | 0.090 | Dnajc21 | 1.24 | 0.184 | Hspa4 | 0.81 | 0.046* |
| Bag1 | 1.05 | 0.287 | Dnajb12 | 0.87 | 0.044* | Dnajc3 | 0.83 | 0.125 | Hspa4l | 1.10 | 0.320 |
| Bag2 | 1.08 | 0.473 | Dnajab13 | 1.42 | 0.135 | Dnajc4 | 0.77 | 0.126 | Hspa5 | 1.04 | 0.551 |
| **Bag3** | **0.72** | **0.044*** | Dnajb14 | 0.94 | 0.353 | Dnajc5 | 0.90 | 0.102 | Hspa8 | 0.96 | 0.702 |
| Bag4 | 1.1 | 0.283 | **Dnajb2** | **0.82** | **0.041*** | Dnajc5b | 1.75 | 0.100 | **Hspa9** | **0.72** | **0.025*** |
| Bag5 | 0.82 | 0.091 | Dnajb5 | 0.95 | 0.627 | Dnajc5g | 1.86 | 0.092 | Hspb1 | 0.36 | 0.003** |
| Ccs | 0.78 | 0.077 | Dnajb6 | 1.04 | 0.575 | Dnajc6 | 1.73 | 0.029* | Hspb2 | 1.16 | 0.527 |
| Cct2 | 0.96 | 0.557 | Dnajb7 | 1.18 | 0.250 | Dnajc7 | 0.91 | 0.268 | Hspb3 | 1.66 | 0.038* |
| Cct3 | 0.87 | 0.095 | Dnajb8 | 1.31 | 0.479 | Dnajc8 | 0.96 | 0.659 | Hspb6 | 0.50 | 0.018* |
| **Cct4** | **0.82** | **0.043*** | Dnajb9 | 0.59 | 0.012* | Dnajc9 | 1.06 | 0.623 | **Hspb7** | **0.38** | **0.026*** |
| Cct5 | 1.12 | 0.271 | Dnajc1 | 0.70 | 0.005** | Hsf1 | 1.58 | 0.331 | Hspb8 | 1.10 | 0.308 |
| Cct6a | 1.06 | 0.491 | Dnajc10 | 0.73 | 0.038* | Hsf2 | 1.22 | 0.065 | **Hspd1** | **0.83** | **0.045*** |
| Cct6b | 1.62 | 0.072 | Dnajc11 | 0.91 | 0.067 | Hsf4 | 1.26 | 0.341 | Hspe1 | 0.90 | 0.065 |
| Cct7 | 0.93 | 0.873 | Dnajc12 | 1.29 | 0.183 | Hsp90aa1 | 0.87 | 0.131 | Hsph1 | 0.96 | 0.588 |
| Cryaa | 1.09 | 0.771 | Dnajc13 | 0.86 | 0.244 | Hsp90ab1 | 1.05 | 0.446 | **Pfdn1** | **0.82** | **0.035*** |
| **Cryab** | **0.51** | **0.007**** | Dnajc14 | 0.95 | 0.442 | Hsp90b1 | 0.98 | 0.855 | Pfdn2 | 0.92 | 0.421 |
| Dnaja1 | 0.95 | 0.509 | Dnajc15 | 1.38 | 0.174 | Hspa14 | 0.89 | 0.468 | **Serpinh1** | **0.83** | **0.021*** |
| Dnaja2 | 1.01 | 0.876 | **Dnajc16** | **0.75** | **0.033*** | **Hspa1a** | **0.51** | **0.005**** | Sil1 | 0.86 | 0.285 |
| **Dnaja3** | **0.61** | **0.022*** | Dnajc17 | 1.01 | 0.733 | **Hspa1b** | **0.47** | **0.030*** | Tcp1 | 1.16 | 0.182 |
| **Dnaja4** | **0.50** | **0.004**** | Dnajc18 | 0.77 | 0.048* | Hspa1l | 0.99 | 0.931 | Tor1a | 1.25 | 0.070 |

mRNA levels of HSPs in WT and PGC1α-null fibroblasts exposed to heat shock (42°C) for 1 hour.

*, P<0.05 and **, P<0.01. Genes with bold fonts highlight common genes that up-regulated with PGC1α overexpression (Supplementary Table 1) and down-regulated in PGC1α KO cells after heat shock (Supplementary Table 2).
