# Supplementary material for: The transcriptional coactivator PGC1α protects against hyperthermic stress via cooperation with the heat shock factor HSF1
Source: Cell Death Dis. 2016 Feb 18;7(2):e2102–. doi: 10.1038/cddis.2016.22 (PMC5399192; doi:10.1038/cddis.2016.22)
Supplement: Supplementary Table 3 [file cddis201622x8.doc]

| Primers | Forward Primer 5'-3' | Reverse Primer 5'-3' |
| --- | --- | --- |
| m36B4 | GCTTCATTGTGGGAGCAGAC | ATGGTGTTCTTGCCCATCAG |
| mPgc1α | ACCATGACTACTGTCAGTCACTC | GTCACAGGAGGCATCTTTGAAG |
| mUcp1 | GGCCCTTGTAAACAACAAAATAC | GGCAACAAGAGCTGACAGTAAAT |
| mUcp2 | ATGGTTGGTTTCAAGGCCACA | TTGGCGGTATCCAGAGGGAA |
| mCytC | AAATCTCCACGGTCTGTTCGG | GGGTATCCTCTCCCCAGGTG |
| mDnaja3 | AACATCCCTGTGTCGCAAG | CACCGGGTCTCAATGTCAGC |
| mDnajc19 | ATAAAGGGAAGATCAGGGATGCT | ACATGGTGGTCTCACATGGTT |
| mHspd1 | CACAGTCCTTCGCCAGATGAG | CTACACCTTGAAGCATTAAGGCT |
| mHspa1a | TGGTGCTGACGAAGATGAAG | AGGTCGAAGATGAGCACGTT |
| mHspa9 | ATGGCTGGAATGGCCTTAGC | ACCCAAATCAATACCAACCACTG |
| mBag3 | CTGGGAGATCAAAATCGACCC | GCTGAAGATGCAGTGTCCTTAG |

**Supplementary Table 3** Primers used in RT-PCR assays
